# Gender-specific regulation of response to thyroid hormone in aging

**DOI:** 10.1186/1756-6614-5-1

**Published:** 2012-01-26

**Authors:** Satoru Suzuki, Shin-ichi Nishio, Teiji Takeda, Mitsuhisa Komatsu

**Affiliations:** 1 Department of Aging Medicine and Geriatrics, Institute on Aging and Adaptation, Shinshu University, Graduate School of Medicine, 3-1-1, Asahi, Matsumoto, 390-8621, Japan

**Keywords:** thyroid hormone, TSH, aging

## Abstract

**Background:**

Similar to other systems, the endocrine system is affected by aging. Thyroid hormone, the action of which is affected by many factors, has been shown to be associated with longevity. The most useful marker for the assessment of thyroid hormone action is TSH level. Although age and gender are believed to modify the pituitary set point or response to free thyroid hormone concentration, the precise age- and gender-dependent responses to thyroid hormone have yet to be reported.

**Methods:**

We analyzed the results of 3564 thyroid function tests obtained from patients who received medication at both out- and inpatient clinics of Shinshu University Hospital. Subjects were from among those with thyroid function test results in the normal or mildly abnormal range. Based on a log-linear relationship between the concentrations of FHs and TSH, we established the putative resistance index to assess the relation between serum FH and TSH levels.

**Results:**

Free thyroid hormone and TSH concentration showed an inverse log-linear relation. In males, there was a negative relationship between the free T3 resistance index and age. In females, although there were no relationships between age and FHs, the indices were positively related to age.

**Conclusions:**

These findings indicated that there is a gender-specific response to thyroid hormone with aging. Although the TSH level is a useful marker for the assessment of peripheral thyroid hormone action, the values should be interpreted carefully, especially with regard to age- and gender-related differences.

## Introduction

In common with other systems, the endocrine system is affected by aging. With regard to thyroid hormone, age modifies the pituitary set point or response to comparably reduced free T4 (FT4) concentrations, resulting in lesser serum TSH elevation in older individuals [1]. TSH is suppressed in elderly subjects [2]. The decreased thyroid hormone levels observed in aging are due to lower TSH concentrations [3]. Centenarians exhibit significantly lower TSH levels together with slightly higher reverse T3 levels than aged controls [4]. In contrast to several reports on the association between low TSH level and senescence, heritable longevity has been reported to be associated with high serum TSH and low free T4 levels [5]. Exceptional longevity is associated with raised serum TSH [6,7]. Thus, the relation between the level of TSH and lifespan is controversial.

TSH concentration is higher in women than in men [8]. TSH daily profiling demonstrated that TSH secretion depends on age in women only [9]. These reports suggest that thyroid hormone function, especially TSH level, may be associated with gender differences.

Both T3 and T4 are often loosely referred to as thyroid hormones, although T3 alone has biological thyromimetic activity at the genomic level. The ratio of serum FT3 concentration to FT4 concentration, [FT3]/[FT4] index, is a useful indicator to assess the peripheral metabolism of thyroid hormone, as the index is affected by the magnitude of the conversion of FT4 to FT3 or of the transport of T4 into T3-producing tissues [10].

Serum concentration of thyroid hormone is inversely related to TSH concentration. Although the serum concentration reflects the action of thyroid hormone in cells, clinical manifestations of thyroid hormone action are occasionally not related to the serum hormone concentration. For example, we observe low FT3 concentrations in patients with severe disorders or infection, *i.e*., *low *T3 syndrome. Moreover, molecular studies have strongly suggested that the nuclear action of thyroid hormone may be affected by numerous factors. MCT8 is a specific transporter of T3 [11]. μ-crystallin is a cytoplasmic thyroid hormone binding protein within cells, which may also affect the action of thyroid hormone [12]. Phosphodiesterase 8B gene variants are associated with serum TSH levels and thyroid function [13]. Molecules that affect the transcriptional machinery also alter the response to thyroid hormone [14]. Taken together, these clinical and molecular findings suggest that factors responsible for the action of thyroid hormone except for thyroid hormone itself are present *in vivo*. The patho-physiological alteration may manifest thyroid hormone resistance, similar to the insulin resistance in diabetic or obese patients.

Free hormones (FHs) are undoubtedly the strongest factors that affect the regulation of TSH expression. There is a log-linear relationship between free thyroxin and TSH over a wide concentration range [15]. Although the resistance factors affect the regulation of TSH secretion, these effects may be diminished in the severe hyper- or hypothyroid state. That is, if the resistance factors exist, these factors may be clearly observed in euthyroid or moderate hyper- and hypothyroid states.

Based on these speculations, we selected data with TSH from 0.04 to 9.99 μIU/ml and FT4 from 0.52 to 2.1 ng/dl. We established the putative resistance index obtained from the serum concentration of thyroid hormone and TSH, and investigated the existence of age- and gender-related factors that affect thyroid hormone resistance. In addition, we discuss the physiological mechanisms of gender-specific and age-oriented thyroid hormone resistance.

## Materials and methods

### Subjects

A total of 1026 male and 2538 female (median age 44 years, range 15 - 92 years) subjects were included in this study. All patients were referred to in- or outpatient clinics of Shinshu University Hospital from January 2004 to January 2005 due to suspected thyroid disease or to receive treatment for various thyroid diseases, including Graves' disease, autoimmune and non-immunogenic thyroiditis, benign diffuse and nodular goiter, and thyroid malignancies. This study was approved by the Ethical Committee of Shinshu University (reference number 818).

### Laboratory methods

Standard laboratory quality evaluation procedures were routinely employed, and regular participation at inter-laboratory tests was also part of the quality management strategy. Inter- and intraassay coefficients of variation were 2.2 and 0.8% for TSH, 4.5 and 1.8% for FT3, 4.6 and 1.3% for FT4, respectively.

### Statistical analysis

For pairs of continuous variables, scatter plots were constructed to visually display the respective relations. If found to be linear with both variables showing a normal distribution, Pearson's correlation was computed to statistically describe the significance, strength, and nature of the relation using SPSS version 18. In the age distribution, statistical significance was determined by *Kruskal-Wallis *followed by the *Games-Howell *multiple comparison test. *P *values ≥ 0.05 were considered not significant.

## Results

### Characteristics of the study population

**Figure 1:**
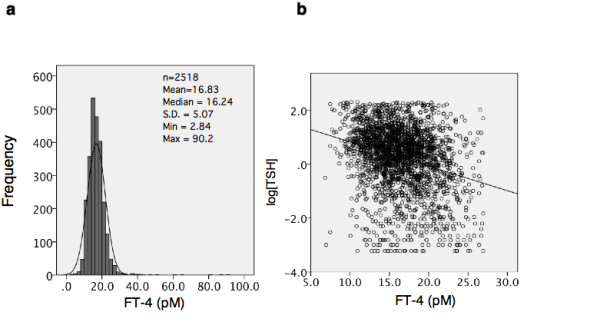
**Frequency distribution of serum free T4 concentration and log-linear relationship of the serum concentration between of free T4 and of TSH in this study**. (a) Frequency distribution of serum free T4 concentration. The gray column represents 2519 samples with TSH ranging from 0.04 to 9.99 selected from the total of 3564 samples. (b) The relationship between [FT4] and log[TSH]. The line represents the linear regression fitted to the data points using SPSS software.

To select the normal and subnormal regulation of thyroid hormone and TSH, we omitted data from subjects with serum TSH concentration ([TSH]) less than 0.04 μIU/ml and greater than 9.99 μIU/ml. We also omitted data from those with serum FT4 concentration ([FT4]) less than 6.7 pM (0.52 ng/dl) and greater than 27.0 pM (2.1 ng/dl). The adopted range was mean ± 2 *S.D*. as shown in Figure 1a. A total of 735 male and 1721 female subjects were included in the analysis. The detailed characteristics of the study population are shown in Table 1.

**Table 1:** Clinical characteristics of the study population

Characteristics	male	female
	*n *= 735	*n *= 1721
Age - years		
Mean ± SD	46.94 ± 16.24	44.05 ± 17.0
Range	15 - 84	15 - 82
Free T3 - pM		
Mean ± SD	5.91 ± 1.7	5.36 ± 1.55
Range	1.38 - 9.97	0.84 - 9.85
Free T4 - pM		
Mean ± SD	16.85 ± 3.81	16.3 ± 3.51
Range	6.96 - 26.9	6.83 - 26.9
TSH - μIU/ml		
Mean ± SD	2.44 ± 2.14	1.99 ± 1.91
Range	0.04 - 9.99	0.04 - 9.95
log [TSH]		
Mean ± SD	0.464 ± 1.06	0.142 ± 1.21
Range	-3.21 - 2.30	-3.21 - 2.30
ResT3		
Mean ± SD	1.22 ± 0.26	1.10 ± 0.27
Range	0.27 - 1.90	0.24 - 1.91
ResT4		
Mean ± SD	1.16 ± 0.25	1.08 ± 0.23
Range	0.05 - 1.78	0.16 - 1.93
[FT3]/[FT4]		
Mean ± SD	0.37 ± 0.13	0.34 ± 0.12
Range	0.11 - 0.84	0.06 - 0.98

Res: resistance index

### Organization of a resistance index

As shown in Figure 1b, the data in this study showed a log-linear relationship between FT4 and TSH (log[TSH] = 1.729 - 0.09[FT4], *P *< 0.001). The linear regression line also showed a good fit to the data points obtained from FT3 and log [TSH] (log[TSH] = 0.429 - 0.034[FT3] *P *= 0.02). We can express the relation between log[TSH] and the concentration of a free hormone [FH] as follows:

$$\text{log}\left[\text{TSH}\right]=\text{b}-\text{a}\left[\text{FH}\right]\left(\text{a},\text{b};\text{ any positive number}\right)$$

To simplify the equation, we set a = 1:

$$\text{log}\left[\text{TSH}\right]\;=\text{b}-\left[\text{FH}\right]$$

We would express a conventional resistance index (Rconv) as follows:

$$\text{Rconv }=\;\left[\text{FH}\right]+\text{log}\left[\text{TSH}\right]$$

To be equivalent to the strength of each factor, such as [FH] and log[TSH], we converted each factor to a value ranging from 0 to 1 as follows:

$$\begin{array}{ccc} \text{R} & =\left(\left[\text{FH}\right]-{\left[\text{FH}\right]}_{\text{min}}\right)\;/\;\left({\left[\text{FH}\right]}_{\text{max}}-{\left[\text{FH}\right]}_{\text{min}}\right) & \\ & +\left(\text{log}\left[\text{TSH}\right]-\text{log}{\left[\text{TSH}\right]}_{\text{min}}\right)\;/\;\left(\text{log}{\left[\text{TSH}\right]}_{\text{max}}-\text{log}{\left[\text{TSH}\right]}_{\text{min}}\right) \end{array}$$

In this study,

$$\begin{array}{c} {\left[\text{FT}3\right]}_{\text{max}}=9.97\text{pM},\;{\left[\text{FT}3\right]}_{\text{min}}=0.84\text{pM},\;{\left[\text{FT}4\right]}_{\text{max}}=\;26.93\text{pM},\;{\left[\text{FT}4\right]}_{\text{min}}=\;6.83\text{pM},\\ \text{log}{\left[\text{TSH}\right]}_{\text{max}}=2.3\text{ log}{\left[\text{TSH}\right]}_{\text{min}}=-3.22. \end{array}$$

Thus, the T3 resistance index (ResT3) and T4 resistance index (ResT4) were defined as follows:

$$\begin{array}{ccc} \text{ResT}3\; & =\;\left(\left[\text{FT}3\right]-0.84\right)/9.13+\left(\text{log}\left[\text{TSH}\right]+3.22\right)/5.52 & \\ \text{ResT}4 & =\left(\left[\text{FT}4\right]-6.83\right)/20.1+\left(\text{log}\left[\text{TSH}\right]+3.22\right)/5.52 \end{array}$$

### Concentration of free thyroid hormone and resistance indices were decreased with aging in males

**Figure 2:**
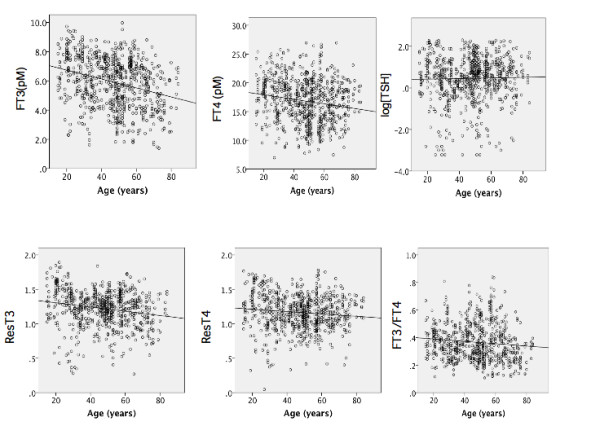
**The relationship between age versus the concentration of FT3 (a), FT4 (b), log[TSH] (c), ResT3 (d), ResT4 (e) and [FT3]/[FT4] index(f) in male subjects**. The line represents the linear regression fitted to the data points calculated by SPSS software.

We performed linear regression analysis between age and concentrations of free T3, T4, and TSH, values of log[TSH], both resistance indices and the [FT3]/[FT4] index. In males, the concentrations of FHs were negatively related to aging, while the TSH level was not (Figure 2a, b and 2c, Table 2). Both resistance indices decreased significantly with age (Figure 2d and 2e). The [FT3]/[FT4] index decreased significantly (Figure 2f, Table 2).

**Table 2:** Spearman's rank correlation coefficient between age and various thyroid hormone indices

*vs*. Age	male	female
	*n *= 735	*n *= 1721
Free T3	-0.292 (0.001>)**	-0.033 (0.174)
Free T4	-0.170 (0.001>)**	0.014 (0.573)
TSH	-0.042 (0.255)	0.138 (0.001>)**
log[TSH]	0.022(0.548)	0.099 (0.001>)**
ResT3	-0.194 (0.001>)**	0.061 (0.012)*
ResT4	-0.111 (0.003)**	0.106 (0.001>)**
FreeT3/FreeT4	-0.102 (0.006)**	-0.025 (0.298)

Res: resistance index Parentheses show *P*-values. *P < 0.05, **P < 0.01.

### In females, the free thyroid hormone concentration was not altered by aging, but the TSH level increased in an age-dependent manner

**Figure 3:**
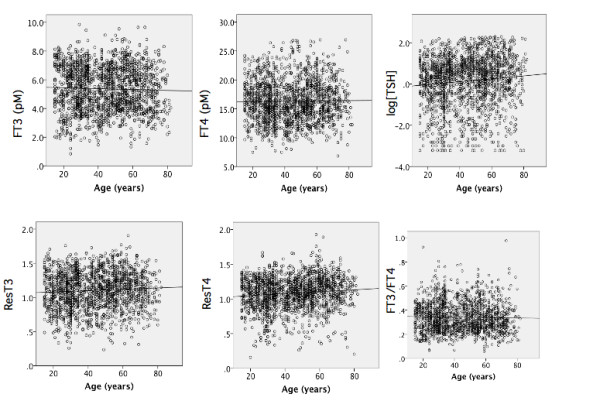
**Relationships between age and the concentration of FT3 (a), FT4 (b), log[TSH] (c), ResT3 (d), ResT4 (e), and [FT3]/[FT4] index (f) in female subjects**. The line represents the linear regression fitted to the data points calculated using SPSS software.

We plotted the values of a series of thyroid hormone indices *vs*. age in females. There were no relationships between the concentrations of both FHs and age (Figure 3a and 3b, Table 2). TSH level was significantly increased (Figure 3c). Both resistance indices increased with age (Figure 3d and 3e). The [FT3]/[FT4] index did not change significantly with age (Figure 3f).

### Gender-specific differences in resistance indices in the age distribution

**Figure 4:**
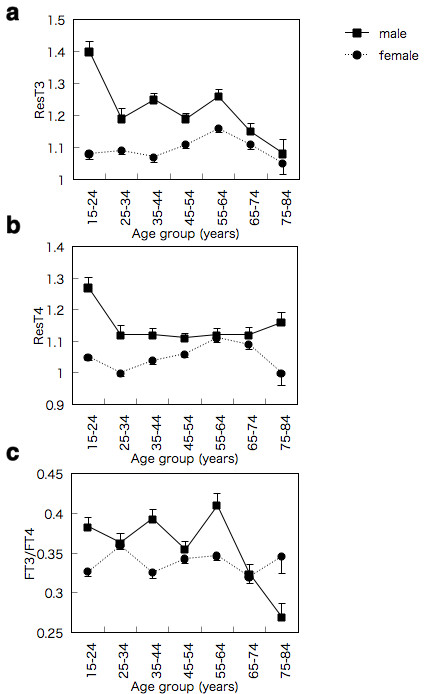
**Age distributions of ResT3 (a), ResT4 (b), and [FT3]/[FT4] index (c) in males and females**. The data represents the means ± *S.E*. of each age group. Bold and broken lines represent the values for males and females, respectively. Statistical significances show in Table 3.

According to our mathematical model, the age-dependent distribution revealed a specific pattern in both male and female subjects. As shown in Figure 4a, the mean values of the T3 index decreased in the age group from 15 - 24 to 25 - 34, and then increased slightly in the 25 - 64 age groups in males. The value decreased again over the 55 - 64 age group in males. The mean values of the T3 index increased gradually between the 35 - 44 and 55 - 64 age groups in females. Although there was no statistical significance, the values were decreased in females over 65.

On the other hand, the mean values of the T4 index were reduced in males between 15 - 24 and 25 - 34 (Figure 4b). The values were constant over the 25 - 34 age group. In females, the mean values of the T4 index initially decreased between 15 - 24 and 25 - 34. The values increased gradually over the 25 - 34 age group except in the 65 - 74 and 75 - 84 age groups. The mean values of the [FT3]/[FT4] index decreased in males in the 65 - 74 and 75 - 84 age groups, while the value remained constant in females (Figure 4c). The statistical differences were shown in Table 3.

**Table 3:** Statistical differences between each age group in male (upper area) and in female (lower area).

Age group	15-24	25-34	35-44	45-54	55-64	65-74	75-84
15-24 ResT3		0.001 > **	0.003**	0.001 > **	0.007**	0.001 > **	0.001 > **

15-24 ResT4		0.011*	0.001**	0.001 > **	0.002**	0.003**	0.189

15-24 FT3/FT4		0.866	0.998	0.441	0.839	0.007**	0.001 >

25-34 ResT3	0.999		0.639	1.0	0.508	0.920	0.358

25-34 ResT4	0.399		1.0	1.0	1.0	1.0	0.969

25-34 FT3/FT4	0.024*		0.557	0.999	0.218	0.251	0.001

35-44 ResT3	1.0	0.992		0.131	1.0	0.028*	0.017**

35-44 ResT4	1.0	0.668		1.0	1.0	1.0	0.914

35-44 FT3/FT4	1.0	0.031*		0.154	0.981	0.001**	0.001 > **

45-54 ResT3	0.778	0.922	0.649		0.072	0.857	0.282

45-54 ResT4	0.980	0.030*	0.875		1.0	1.0	0.764

45-54 FT3/FT4	0.787	0.416	0.796		0.046*	0.383	0.001 > **

55-64 ResT3	0.011*	0.013*	0.006**	0.331		0.016*	0.012*

55-64 ResT4	0.011*	0.001 > **	0.004**	0.074		1.0	0.913

55-64 FT3/FT4	0.622	0.780	0.635	1.0		0.001 > **	0.001 > **

65-74 ResT3	0.827	0.947	0.717	1.0	0.432		0.825

65-74 ResT4	0.175	0.001 > **	0.083	0.541	0.985		0.939

65-74 FT3/FT4	0.997	0.006**	0.998	0.433	0.301		0.165

75-84 ResT3	0.995	0.959	0.998	0.715	0.102	0.738	

75-84 ResT4	0.957	1.0	0.985	0.834	0.189	0.379	

75-84 FT3/FT4	0.992	0.995	0.991	1.0	1.0	0.958	

*P < 0.05, **P < 0.01.

## Discussion

In this study, we selected the cases with normal or subnormal thyroid function. Despite the narrow range of the study population, an inverse log-linear relation between FH and TSH was observed as described previously [16]. Benhadi *et al*. denoted the straight line as the set point of the hypothalamus-pituitary-thyroid axis [17]. The set point was probably affected by many factors, including genetic and environmental factors.

In this study, when we include the samples whose TSH concentration is less than 0.04 μIU/ml, the value of 2 *S.D *is larger than the mean value in free T4 concentration. Thus we omitted the samples whose TSH concentration is less than 0.04 μIU/ml. There is no mathematical reason why we omitted the samples whose TSH concentration is over 9.99 μIU/ml. We should note that subclinical hypothyroidism is associated with an increased risk of cardiovascular disease events and mortality in those with higher TSH levels, particularly in those with TSH concentration of 10 μIU/ml or greater [18].

In male subjects, negative relationships were observed between aging and serum thyroid hormone concentration. TSH level was not altered by aging. Both resistance indices were suppressed by aging. These data indicated that the age-dependent lower FH concentrations do not induce a compensatory increase in TSH expression in males. There are two possible explanations for these results. One is that age-dependent suppression of TSH secretion is latently present, as described previously [1]. As a result, the lower serum hormone-induced TSH expression may not overcome the suppressed TSH secretion. The other possibility is that lower serum hormone may be sufficient to suppress TSH secretion because the resistance indices are suppressed. It is possible that males show age-dependent sensitization of thyroid hormone action.

Aging did not affect the serum concentrations of free thyroid hormones in female. The TSH levels increased in an aging-dependent manner. Both resistance indices increased significantly with age. These data indicated that age-dependent thyroid hormone resistance in females in contrast to the sensitization in males. The ratio of [FT3] to [FT4] was reduced by aging in males but not in females, suggesting that the deiodination and/or transport of T4 were decreased in males.

The precise age distribution suggests that there are three phases in the lifespan in terms of thyroid hormone resistance. The first phase is the age between 15-24. The values of both indices were suppressed in male, suggesting that some unknown factors may affect the response to thyroid hormone in adolescent male. It is possible that testosterone plays roles in regulation of response to thyroid hormone, since free dihydrotestosterone concentration peaks at the age group 20-25 [19]. The second phase is the age between 25-64 years old. The mean values of both T3 and T4 indices were not altered in males aged between 25 - 34 and 55 - 64. There was, however, a tendency for the resistance indices to increase in the same 25 - 64 age groups, the pre-menopausal phase in females. Many studies have suggested that estrogen may overlap the thyroid hormone action [20]. These data taken together indicate that age-dependent depletion of estrogen may contribute to the progression of resistance to thyroid hormone action in females.

The third phase is over 64 years old. The mean values of the T3 resistance index were suppressed in males over 64 years old, while that of the T4 index was not significantly altered in both genders. The [FT3]/[FT4] index was also decreased in males over 64, but not in females, suggesting that the lack of a reduction in the T4 index in males is probably due to the impaired conversion of FT4 to FT3. In females, both resistance indices were not increased although the reasons remain unclear.

There are two limitations in this study. One is that the subject is heterogeneous in terms of treatment. Obviously, some patients may be administered supplemental T4 or anti-thyroid medication and/or radioiodine therapy. These modifications may somehow affect thyroid function, especially the conversion of FT4 to FT3. These biases may affect the results of the [FT3]/[FT4] index. Although we omitted the data from subjects with extremely low TSH, we may not have excluded patients with mild or moderate hypopituitarism. It is, however, difficult to discriminate physiological hypopituitarism from pathological disorders, as described previously [3]. We consider that age-related pituitary-thyroid regulation may not be affected by pathological thyroid diseases, since the accumulation of vulnerability with age is an all around and non-specific process as described [21]. It is assumed that serum thyroid hormone is the most potent factor to regulate TSH level in all age groups. As both FH and TSH concentrations were normal or mildly abnormal range in the groups, TSH preferentially regulates FH secretion vice versa at the time of sampling the serum. Thus, the data selected in this study were likely appropriate for assessment of negative feedback regulation of thyroid hormone in aging.

Another issue is that the reflex indices in this study may not precisely reflect the strength of the resistance to thyroid hormone. Mathematically, there is a report that log [TSH] is negatively proportional to the square of free T4 concentration [22]. Since there is negative linear relationship between log [TSH] and free concentrations, we conveniently added the two factors after modification of the strength in this study.

In conclusion, we demonstrated the age-dependent sensitization and resistance to thyroid hormone in males and females, respectively. Due to the complexity of negative feedback regulation, TSH level alone could not be used to assess the thyroid hormone action *in vivo*. As TSH level is a convenient marker for assessment of thyroid hormone action, it is necessary to exercise care in interpretation of the values, especially on the basis of age- and gender-related insights. The precise physiological significance of gender-specific resistance to thyroid hormone remains to be elucidated.

## Abbreviations

FH: free hormone; FT3: free T3; FT4: free T4; ResT3: T3 resistance index; ResT4:T4 resistance index.

## Competing interests

The authors declare that they have no competing interests.

## Authors' contributions

SN collected and summarized the data obtained from individual patients. TT and MK contributed to discussion and edited the manuscript. SS conceived of the experimental design, analyzed data and wrote the manuscript. All authors read and approved the final manuscript.
